# In breast carcinoma dysadherin expression is correlated with invasiveness but not with E-cadherin

**DOI:** 10.1038/sj.bjc.6603743

**Published:** 2007-04-17

**Authors:** A Batistatou, D Peschos, H Tsanou, A Charalabopoulos, Y Nakanishi, S Hirohashi, N J Agnantis, K Charalabopoulos

**Affiliations:** 1 Department of Pathology, University of Ioannina Medical School, P.O. Box 1186, Ioannina 45110, Greece; 2 Department of Surgery, Peterborough and Stamford Hospitals, Cambridgeshire, UK; 3 Pathology Division, National Cancer Center Research Institute, Tokyo, Japan; 4 Department of Physiology, Clinical Unit, University of Ioannina Medical School, P.O. Box 1186, Ioannina 45110, Greece

**Keywords:** dysadherin, E-cadherin, breast, ductal carcinoma, lobular carcinoma

## Abstract

Reduction/loss of E-cadherin is associated with the development and progression of many epithelial tumours. Dysadherin, recently characterised by members of our research team, has an anti-cell–cell adhesion function and downregulates E-cadherin in a post-transcriptional manner. The aim of the present study was to study the role of dysadherin in breast cancer progression, in association with the E-cadherin expression and the histological type. We have selected ductal carcinoma, which is by far the most common type and lobular carcinoma, which has a distinctive microscopic appearance. Dysadherin and E-cadherin expression was examined immunohistochemically in 70 invasive ductal carcinomas, no special type (NST), and 30 invasive lobular carcinomas, with their adjacent *in situ* components. In ductal as well as in lobular carcinoma dysadherin was expressed only in the invasive and not in the *in situ* component, and this expression was independent of the E-cadherin expression. Specifically, all 10 (100%) Grade 1, 37out of 45(82.2%) Grade 2 and six out of 15 (40%) Grade 3 invasive ductal carcinomas showed preserved E-cadherin expression, while ‘positive dysadherin expression’ was found in six out of 10 (60%) Grade 1, 34 out of 45(75.5%) Grade 2 and all 15 (100%) Grade 3 neoplasms. None of the 30 infiltrating lobular carcinomas showed preserved E-cadherin expression, while all the 30 infiltrating lobular carcinomas exhibited ‘positive dysadherin expression’. Dysadherin may play an important role in breast cancer progression by promoting invasion and, particularly in lobular carcinomas, it might also be used as a marker of invasion.

Recent advances into molecular pathology of breast cancer have refined diagnostic accuracy and classification systems of the most common malignant neoplasm of women, rendering personalised therapy more possible. Today, there is a plethora of molecular genetic data that indicate differences in pathogenesis between the various types of breast carcinomas and thus support their categorisation, to the patient benefit. The demonstration of lack of E-cadherin expression in lobular neoplasms has had a sound impact with practical applications (Mastracci *et al*, 2005) In about half of lobular carcinomas, loss of E-cadherin involves genetic changes, that is loss of heterozygosity (LOH) at 16q22.1, while in the other half epigenetic events are involved (Knudsen and Wheelock, 2005; Mastracci *et al*, 2005). Ductal carcinomas, on the other hand, express E-cadherin, albeit in reduced levels and/or in abnormal cellular locations (Knudsen and Wheelock, 2005). Reduction/loss of E-cadherin has been associated with the development and progression of many epithelial neoplasms. Aberrant E-cadherin expression (heterogeneous, cytoplasmic, or absent) has been detected immunohistochemically in several cancers, including head and neck carcinoma, gastric adenocarcinoma, lobular breast carcinoma, lung cancer, colorectal carcinoma, prostate adenocarcinoma, pancreatic, and bladder cancer (Becker *et al*, 1994; Hirohashi, 1998; Chang *et al*, 2002; Charalabopoulos *et al*, 2002, 2004; Hirohashi and Kanai, 2003; Mastracci *et al*, 2005; Massarelli *et al*, 2005). In the vast majority of these neoplasms such expression has been associated with poor differentiation, increased metastatic potential and poor prognosis. In breast the scenario is more complicated, since lobular carcinoma, that typically does not express E-cadherin has a more favourable outcome than ductal carcinoma, which in general expresses E-cadherin. Furthermore, there are contradictory data on the possible association between E-cadherin expression and high-grade tumours with increased metastatic potential (Oka *et al*, 1993; Charpin *et al*, 1997; Heimann *et al*, 2000; Gillett *et al*, 2001; Parker *et al*, 2001; Elzagheid *et al*, 2002; Gupta *et al*, 2003; Knudsen and Wheelock, 2005; Rakha *et al*, 2005). It is clear that there are pieces missing from the puzzle of adhesion molecules and breast carcinoma.

Recently, the cloning and characterisation of dysadherin (FXYD5), a cell membrane glycoprotein that has an anti-cell–cell adhesion function and downregulates E-cadherin in a post-transcriptional manner has been reported, by members of our research team. This novel cancer-associated protein has been detected in head and neck, tongue, oesophageal, gastric, colorectal, testicular, pancreatic, thyroid, and cervical carcinomas as well as in malignant melanoma and has been associated with tumour aggressiveness (Ino *et al*, 2002; Tsuiji *et al*, 2002; Aoki *et al*, 2003; Sato *et al*, 2003; Shimamura *et al*, 2003, 2004; Nakanishi *et al*, 2004; Shimada *et al*, 2004a, 2004b; Wu *et al*, 2004; Batistatou *et al*, 2005; Nishizawa *et al*, 2005; Batistatou *et al*, 2006; Kyzas *et al*, 2006). A recent *in vitro* study has demonstrated that dysadherin has prometastatic effects that are independent of E-cadherin expression (Nam *et al*, 2006). The aim of the present study was to investigate further the expression of dysadherin in breast carcinoma, with particular emphasis to the acquisition of a lobular or a ductal phenotype, in combination with E-cadherin expression.

## MATERIALS AND METHODS

One hundred formalin-fixed, paraffin-embedded archival tissue blocks of breast carcinomas were included in the current study and represented an equal number of female patients (mean age 54.5 years, range 35–79). The material consisted of 70 invasive ductal carcinomas, no special type, NST (10 Grade 1, 45 Grade 2 and 15 Grade 3, graded using the modified Bloom and Richardson method), in 30 of which an adjacent *in situ* ductal carcinoma was identified, and 30 invasive lobular carcinomas, in 15 of which an adjacent *in situ* lobular carcinoma was identified.

### Immunohistochemistry

We performed immunostaining on formalin-fixed, paraffin-embedded tissue sections using the EnVision System (DAKO Corp., Netherlands), and the monoclonal antibodies: NCC-M53 against dysadherin and E-cadherin (CM170B, Biocare Medical, CA, USA). Briefly, 4-*μ*m-thick tissue sections were deparaffinised in xylene; rehydrated through graded concentrations of alcohol and heated in a microwave oven for two cycles of 15 min each at 300 W, in citrate buffer, for antigen retrieval. Endogenous peroxidase activity was blocked with H_2_O_2_ solution in methanol (0.01 M), for 30 min. After washing with phosphate-buffered saline (PBS) for 5 min, the primary antibodies NCC-M53 (dilution 1 : 1000) and CM170B (dilution 1 : 50) were applied for incubation (30 min at room temperature and overnight at 4°C respectively). Then the slides were washed for 10 min with PBS and were visualised with the EnVision system using diaminobezidine tetrahydrochloride as a chromogen. Finally, all sections were counterstained with haematoxylin. Positive staining of endothelial cells and lymphocytes was used as an internal positive control for dysadherin. As an internal positive control for E-cadherin, positive staining of non neoplastic ductal epithelial cells was used. As a negative control the first antibody was substituted with normal mouse immunoglobulin of the same class.

### Evaluation of the staining

For each sample, at least 1000 neoplastic cells were counted, and the percentage of cancer cells with positive membranous immunostaining as well as the staining intensity were recorded. For the purposes of statistical analysis, as described previously (Shimada *et al*, 2004b; Batistatou *et al*, 2005), when more than 50% of tumour cells were stained for dysadherin, the tumour was evaluated as ‘positive dysadherin expression (Dys(+))’. When less than 50% of tumour cells were stained for dysadherin, the tumour was evaluated as ‘negative dysadherin expression (Dys(−))’. Regarding E-cadherin, when more than 50% of tumour cells showed complete membranous staining, the tumour was evaluated as ‘preserved E-cadherin expression (E-cad(+))’, while when less than 50% of tumour cells were positive, the tumour was evaluated as ‘reduced E-cadherin expression (E-cad(−))’. Cytoplasmic immunostaining was considered as aberrant expression and was not included in the immunopositive cases.

### Statistical analysis

Analyses were conducted in SPSS software version 11.0 (SPSS, Inc, Chicago, IL, USA). For comparisons between antibodies’ expression with clinicopathological variables we used the *χ*^2^ test. The level of statistical significance was *P*<0.05.

## RESULTS

### Ductal carcinoma

**Figure 1:**
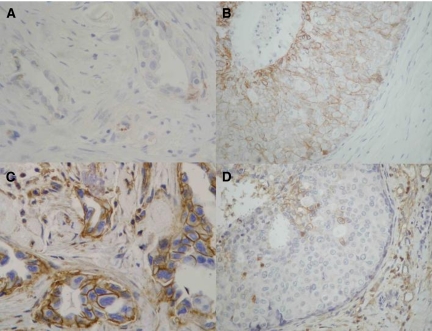
A case of invasive ductal carcinoma, grade II, with adjacent *in situ* component. (**A**) E-cadherin expression is significantly reduced in invasive ductal carcinoma (DABX400). (**B**) Membranous expression of E-cadherin is retained in the adjacent *in situ* component (DABX400). (**C**) Strong, membranous expression of dysadherin is evident in invasive ductal carcinoma (DABX400). (**D**) Dysadherin is not expressed in the adjacent *in situ* component (DABX400).

Membranous E-cadherin expression was detected in epithelial cells of non-neoplastic ducts and acini and this served as internal positive control. In neoplastic cells there was some variation in distribution, depending on the grade and the pattern of stroma infiltration. Specifically, all 10 (100%) Grade 1, 37 out of 45 (82.2%) Grade 2 and six out of 15 (40%) Grade 3 neoplasms showed preserved E-cadherin expression (Table 1, Figure 1A). In immunopositive Grade 2 and Grade 3 tumours the expression of E-cadherin was more heterogeneous, with variations in intensity and distribution of positive cells. Thus, cells in clusters or in tubular structures exhibited higher percentage and more intense membranous staining than individual cells infiltrating the stroma. In the periphery of the invasive ductal carcinoma an intraductal component was observed in several cases. In this *in situ* ductal component the expression of E-cadherin was similar to the non-neoplastic epithelial cells, homogeneous and stronger that the adjacent invasive component (Figure 1B).

Dysadherin expression was detected in myoepithelial cells of ducts and acini, but not in non-neoplastic epithelial cells, as well as in endothelial cells of vessels and lymphocytes, as described previously (Batistatou *et al*, 2005, 2006). Dysadherin immunostaining was observed in the membranes of the neoplastic cells and it was heterogeneous throughout the neoplasm (Figure 1C). In particular, preferential expression in diffuse than in compact infiltrative areas was detected. Overall, ‘positive dysadherin expression’ was found in six out of 10 (60%) Grade 1, 34 out of 45 (75.5%) Grade 2 and all 15 (100%) Grade 3 neoplasms (Table 1, Figure 1B). Interestingly, in the adjacent *in situ* ductal carcinoma a small proportion of neoplastic cells (<10%) exhibited membranous immunostaining for dysadherin (Figure 1D). Dysadherin expression was not correlated with E-cadherin expression in IDC (*P*>0.05).

### Lobular carcinoma

**Figure 2:**
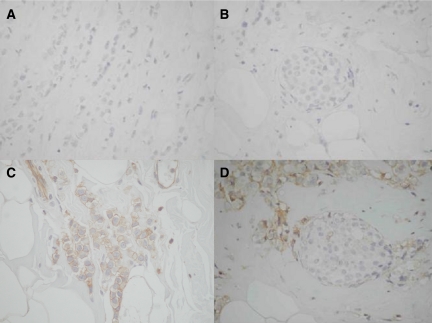
A case of invasive lobular carcinoma, with adjacent *in situ* component. (**A**) E-cadherin expression is lost in invasive lobular carcinoma (DABX400). (**B**) E-cadherin expression is lost in the adjacent *in situ* component (DABX400). (**C**) Membranous expression of dysadherin is evident in invasive lobular carcinoma, (DABX400). (**D**) Dysadherin is not expressed in the adjacent *in situ* component, in contrast with the infiltrating tumour (DABX400).

None of the 30 infiltrating lobular carcinomas showed preserved E-cadherin expression (Table 1, Figure 2A). The vast majority was completely negative, while only in two of them, <20% of neoplastic cells showed weak membranous and cytoplasmic immunopositivity. Interestingly, the adjacent *in situ* lobular carcinoma was completely negative, as well (Figure 2B).

All the 30 infiltrating lobular carcinomas exhibited ‘positive dysadherin expression’ (Figure 2C). In this *in situ* lobular component the expression of dysadherin was limited to a small proportion (<10%) of neoplastic cells (Figure 2D).

## DISCUSSION

Two of the most important characteristics of neoplastic cells are their abilities to grow locally and to metastasise. For both of these processes tumour cells must initially dissociate from each other, either singly or in small nests and invade the surrounding stroma. Today it is generally accepted that at least for carcinomas, adhesion molecules, in particular E-cadherin, play a pivotal role in this process by being downregulated (Hirohashi, 1998; Charalabopoulos *et al*, 2002; Hirohashi and Kanai, 2003). In general, there is an association between aberrant E-cadherin expression, tumour dedifferentiation and poor clinical outcome.

Regarding breast cancer, E-cadherin expression varies depending on the histological subtype. Thus, in ductal carcinoma E-cadherin is expressed, albeit in reduced levels and aberrant cellular locations. Although E-cadherin correlates inversely with the grade of the tumour, reduced E-cadherin expression is not adequate to predict clinical outcome and there are contradictory studies on the association between E-cadherin and survival (Knudsen and Wheelock, 2005). Moreover, it has been reported that in other breast cancers with known poor prognosis, such as inflammatory breast cancer, there is overexpression of E-cadherin (Knudsen and Wheelock, 2005). An interesting concept is that possibly the reduction of E-cadherin expression in breast carcinomas, other than lobular, is transient, due to epigenetic modifications. Several mechanisms for reversible reduction of E-cadherin expression in human neoplasms have been reported (Hirohashi, 1998; Charalabopoulos *et al*, 2002; Hirohashi and Kanai, 2003). Among them, recently, a novel cell membrane glycoprotein named ‘dysadherin’ (from the Greek prothema dys-, which means difficulty, or aberration, or reversibility) has been shown to downregulate E-cadherin in a post-transcriptional manner and reduce cell–cell adhesiveness in *in vitro* studies and in animal models. Dysadherin is a member of the FXYD family (FXYD5 or Related to Ion Channel). It is located at chromosome 19 and has a single transmembrane domain. It interacts with and modulates the properties of the Na^+^, K^+^ ATPase (Ino *et al*, 2002; Lubarski *et al*, 2005). In human tissues increased dysadherin expression has been correlated with the development of metastasis and poor prognosis in gastric, pancreatic, colorectal, oesophageal, thyroid, tongue and cervical carcinomas, as well as in malignant melanoma (Aoki *et al*, 2003; Sato *et al*, 2003; Shimamura *et al*, 2003, 2004; Nakanishi *et al*, 2004; Shimada *et al*, 2004a, 2004b; Wu *et al*, 2004; Batistatou *et al*, 2005, 2006; Nishizawa *et al*, 2005; Kyzas *et al*, 2006). Furthermore, in a small pilot series of breast cancer patients, dysadherin expression was correlated with poor prognosis (Ino *et al*, 2002). In most of these neoplasms, as well as in testicular tumours and lymph node metastases of colorectal adenocarcinoma, increased dysadherin expression was correlated with reduced E-cadherin expression (Aoki *et al*, 2003; Sato *et al*, 2003; Shimamura *et al*, 2003, 2004; Wu *et al*, 2004; Batistatou *et al*, 2005, 2006). In invasive ductal carcinoma, as reported in this study, there is an increase in dysadherin expression, which is not related to E-cadherin expression. This lack of association has also been reported in pancreatic, primary colorectal and gastric carcinomas (Shimamura *et al*, 2003; Shimada *et al*, 2004b; Batistatou *et al*, 2005). Furthermore, in the *in situ* ductal carcinoma dysadherin was not expressed. On the basis of these data we would like to propose that in ductal carcinomas, dysadherin can promote invasion independently of the E-cadherin expression.

Lobular breast carcinomas, typically exhibit loss of E-cadherin expression, but they tend to have a more favourable clinical outcome than the more common ductal carcinomas. This loss is an early event affecting not only lobular carcinoma *in situ* but even atypical lobular neoplasia (Mastracci *et al*, 2005). This silencing of E-cadherin is attributed to genetic as well as epigenetic events (Knudsen and Wheelock, 2005; Mastracci *et al*, 2005). In approximately 50% of lobular carcinomas loss of E-cadherin involves LOH at the chromosomal region of 16q, which includes the E-cadherin gene CDH1 locus and mutations in the remaining allele (Kanai *et al*, 1994; Vos *et al*, 1997; Huiping *et al*, 1999; Knudsen and Wheelock, 2005; Mastracci *et al*, 2005). This LOH definitely accompanies mutations in cases of invasive lobular carcinoma, however, the classic pattern of LOH coupled with inactivating mutations in lobular carcinoma *in situ* has not been confirmed. In the other 50% loss of E-cadherin is attributed to epigenetic events, with hypermethylation of the E-cadherin promoter region at CpG islands being one of the most important ones and possibly occurring very early, even at the stage of atypical lobular hyperplasia (Sarrio *et al*, 2004; Shibata *et al*, 2004; Knudsen and Wheelock, 2005; Mastracci *et al*, 2005).

In this study we have confirmed the loss of E-cadherin expression, in *in situ* and invasive breast carcinoma. On the basis of the rare expression of dysadherin in lobular carcinoma *in situ* we can conclude that dysadherin is not responsible for E-cadherin downregulation in lobular carcinoma. An interesting finding from our study is the difference in dysadherin expression between *in situ* and invasive lobular carcinoma. In breast carcinoma the progression from *in situ* to invasive disease is not clearly defined and the specific events that mark the transition to an invasive tumour are under intense investigation. Lack of E-cadherin expression cannot be associated with an invasive phenotype, since it is also evident in the *in situ* component. On the other hand, we have shown that dysadherin is specifically and constantly expressed in invasive lobular carcinomas. On the basis of this finding we propose that dysadherin is a possible causative player in the process of acquiring an invasive phenotype, as well as a possible marker for invasiveness. It has also been proposed that loss of E-cadherin expression is responsible for the distinct pattern of invasion observed in lobular neoplasms (Knudsen and Wheelock, 2005; Mastracci *et al*, 2005). We would like to add that another major contributor to this characteristic invasion pattern, with single cells arranged in cords is the expression of dysadherin. The latter possibly acts, either alone or in conjunction with loss of E-cadherin, by allowing cells to dissociate from each other. Studies on the function of dysadherin are available by experimental data, where dysadherin appears to play an important role in neoplastic cell invasion and metastasis (Ino *et al*, 2002; Nam *et al*, 2006). The exact molecular mechanisms of these effects have not been elucidated yet. Recently, it has been shown that, besides downregulating E-cadherin, dysadherin can promote invasion at least in breast cancer cells *in vitro*, through an E-cadherin-independent mechanism. This mechanism involves enhanced signaling through the NF-*κ*B pathway, which leads to increased production of the tumour-promoting (C-C motif) ligand 2 (CCL2) (Nam *et al*, 2006).

In conclusion, in this study we have investigated the role of specific adhesion/dysadhesion molecules in the development of breast carcinoma. We have selected ductal carcinoma which is by far the most common type, and lobular carcinoma which has a distinctive microscopic appearance. We have shown similarities and differences between these two types. Interestingly, in ductal as well as in lobular carcinoma, dysadherin was expressed only in the invasive and not in the *in situ* component, and this expression was independent of E-cadherin. Thus, dysadherin may play an important role in breast cancer progression by promoting invasion and, particularly in lobular carcinomas, it might also be used as a marker of invasion.

## Figures and Tables

**Table 1:** E-cadherin and dysadherin expression in invasive breast carcinomas

**Histologic type**	**Preserved E-cadherin expression**	**‘Positive’ dysadherin expression**
*Invasive ductal carcinoma*		
Grade 1 (10)	10 (100 %)	6 (60%)
Grade 2 (45)	37 (82.2%)	34 (75.5%)
Grade 3 (15)	6 (40%)	15 (100%)
Total (70)	53 (75.7%)	55 (78.6%)
		
*Invasive lobular carcinoma*		
Total (30)	0 (0%)	30 (100%)
